# Book Review

**Published:** 1908-09

**Authors:** 


**The Technique of Vagino-Peritoneal Operations.** By E. WERTHEIM and TH. MICHOLITSCH. Translated by CUTHBERT LOCKYER, M.D. Pp. 323. London: Macmillan and Co., Limited. 1907.—This work is of the greatest possible value to all those who are in the habit of operating on the pelvic organs by the vaginal route. It gives a whole series of extremely clear and excellent illustrations from photographs of the various steps in the operations of anterior and posterior colpotomy, which are delineated with great care and very clearly. They will be found most valuable by those who practise these operations, whether they are commencing or whether they have had experience of operating by this route. We feel no doubt that the range of usefulness of operations by this method is very much greater than has hitherto been supposed, but at the same time there are certain difficulties, mainly of manipulation, to be encountered which put a limit to the use of the vagina for operations on the pelvic organs. For example, the evacuation and removal of ovarian cysts by this route, in the absence of adhesions and by the method indicated, is a matter of no great difficulty; but, at the same time, the presence of adhesions would increase the difficulty very materially, and in our view render this method inferior to that by laparotomy. The same applies to a certain extent to the operation of myomectomy and removal of the uterus, where the latter is subject to fibroids. We have ourselves found that it is perfectly possible to perform such operations as myomectomy or the removal of the ovaries by this route, and consider that the plates and descriptions are of the greatest possible assistance. The full detailed account of Suchardt's operation or the paravaginal section is given, and this method also is one we can recommend as a very valuable adjuvant to the performance of operations by the vaginal route in cases in which this is preferable on account of the patient's general condition, and when at the same time room for manipulative procedures is insufficient. There can be no doubt that the vaginal route is one which is most valuable in cases of operation on elderly and feeble patients where it is wished to avoid shock, but at the same time it not infrequently happens that in such cases room for manipulative procedures is very limited. Suchardt's method will be found to give ample room, and the procedure to be adopted is clearly and simply described and illustrated. We congratulate the translator on his successful presentation of an extremely valuable and instructive work.

**Rotunda Practical Midwifery.** By E. HASTINGS TWEEDY, M.D. and G. T. WRENCH, M.D. Pp. xix., 464. London: Henry Frowde and Hodder and Stoughton. 1908.—This book shows up two points very clearly. First, that it is written by one accustomed to teach; and, secondly, by one accustomed to practise. It is therefore not only sound in principles, but also easy to understand, and essentially practical. The diagnosis of pregnancy, taken month by month, gives one at a glance the conditions to be looked for. The chapter on "Abortion and Miscarriage" is one which will be useful to general practitioners. On a subject such as this, which is really a question of practical details, one generally finds a book singularly deficient, and relies on what cases have come under observation before, or simply, and often soundly, on common sense. Here the book carries one to the bedside, and takes all possibilities as they arise. The necessity for having a thorough grasp of mechanisms in all conditions does not seem to have impressed the authors; their teaching on this head is scanty, involved, and the sketches not always convincing. The only other fault we would find is the very short space given to general diseases in pregnancy. For instance, heart disease is dismissed in eight lines. The book is one we would advise practitioners to buy.

**Rotunda Midwifery for Nurses and Midwives.** By G. T. WRENCH, M.D. Pp. xiv., 324. London: Henry Frowde and Hodder and Stroughton. 1908.—This little work is so much on the lines of the larger one—*Rotunda Practical Midwifery*—that we need say but little more about it. The style, teaching and diagrams are largely identical. The Appendix is useful, pointing out clearly the relation of the midwife to the State.

**A Text-Book of Treatment.** Edited by R. J. FERGUSON, M.D. Part I.—"Obstetrics and Gynecology." By JOHN CAMPBELL, M.A., M.D. Pp. xii., 279. London: Sidney Appleton. 1908.—We cannot think for whom these alphabetical books are written. If there is a demand for them, then we suppose it must serve as an excuse for the editor. We looked for "Mechanisms" under that name, under "Labour," under "Presentations," but failed to find them. Taking two others at random, there is nothing under "Antiseptics" or "Cæsarean" Section. The borrowed illustrations are very good.

